# Process Optimization of Thawed Cloudy Huyou Juice Clarification Using a Composite of Carboxymethyl Chitosan and Sodium Alginate

**DOI:** 10.3390/foods14152658

**Published:** 2025-07-29

**Authors:** Peichao Zhang, Liang Zhang, Xiayu Liu, Yuxi Wang, Jiatong Xu, Pengfei Liu, Boyuan Guan

**Affiliations:** 1 College of Biosystems Engineering and Food Science, Zhejiang University, Hangzhou 310058, China; zhangpc0317@163.com (P.Z.); 12213055@zju.edu.cn (Y.W.); jtxu@zju.edu.cn (J.X.); 18838918860@163.com (P.L.); 2 Innovation Center of Yangtze River Delta, Zhejiang University, Jiaxing 314100, China; zlcauer@126.com (L.Z.); xiayuliu@zju.edu.cn (X.L.); 3 Zhejiang Key Laboratory of Functional Structural Lipid Synthesis and Application, Jiaxing 314100, China

**Keywords:** huyou juice, clarification, carboxymethyl chitosan, sodium alginate, response surface methodology

## Abstract

Cloudy huyou juice is increasingly popular for its unique flavor, but flocculent precipitation after cold storage and thawing affects its sensory quality and increases production costs. This study optimized the clarification of thawed cloudy huyou juice using a composite of carboxymethyl chitosan (CC) and sodium alginate (SA), prepared via ionic and covalent crosslinking. The composite was characterized by SEM, FTIR, and thermal analysis. Transmittance was used to evaluate clarification performance. The effects of dosage, adsorption time, and temperature were first assessed through single-factor experiments, followed by optimization using a Box–Behnken response surface methodology. The composite significantly improved clarity (*p* < 0.05), reaching 85.38% transmittance under optimal conditions: 22 mg dosage, 80 min time, and 38 °C. The composite dosage and temperature were the most influential factors. Reusability tests showed declining performance, with the transmittance dropping to 57.13% after five cycles, likely due to incomplete desorption of adsorbed compounds. These results suggest that the CC-SA composite is an effective and reusable clarifying agent with potential for industrial applications in turbid fruit juice processing.

## 1. Introduction

Huyou (*Citrus changshanensis*), derived from a local citrus variety native to Zhejiang Province in China, is well known for its unique flavor, refreshing taste, and potential health benefits [1,2]. Compared to other fruit juices, such as apple or grape juice, huyou juice possesses strong antioxidant and anti-inflammatory properties, which are largely attributed to its richness in bioactive compounds such as nootkatone, scopone, nobiletin, naringin, hesperidin, neohesperidin, limonin, and nomilin [3,4]. These phytochemicals not only contribute to the fruit’s distinctive sensory profile but also enhance its value as a functional beverage. With growing consumer demand for high-quality and functional fruit beverages, cloudy huyou juice (CHJ) has attracted increasing attention in both domestic and international markets [5]. However, during industrial production and cold-chain distribution, CHJ often undergoes flocculation upon thawing from frozen storage. This undesirable phenomenon results in the formation of suspended precipitates, mainly composed of protein–polyphenol or protein–polysaccharide aggregates [6,7], which can significantly compromise the visual appearance, mouthfeel, and overall sensory acceptance of the juice. Moreover, such physical instability shortens huyou juice’s and elevates downstream processing costs, thereby limiting its commercial viability [8].

Previous studies have indicated that flocculation in thawed cloudy juices, including thawed cloudy huyou juice (TCHJ), is primarily driven by non-covalent interactions between proteins and haze-active small molecules, such as polyphenols and pectins. These interactions, governed by electrostatic attraction and hydrogen bonding, contribute to the aggregation and precipitation of colloidal particles [6]. Therefore, it is essential to develop effective clarification strategies capable of selectively removing or neutralizing haze-active substances without impairing the nutritional or organoleptic quality of the juice [9].

Among the various clarification technologies available, the use of natural biopolymer-based clarifying agents has emerged as a promising approach, owing to their safety, biodegradability, and mild processing requirements [10,11]. Sabina et al. investigated the effects of various polysaccharide-based clarifying agents and reaction times on the turbidity of chokeberry juice, and they found that agar-agar (6.1 NTU), carboxymethyl cellulose (6.9 NTU), and xanthan gum (10.0 NTU) achieved the lowest turbidity values after 16 h of treatment [12]. In comparison with single-component clarifiers, composite materials possess more intricate charge distributions and interconnected network structures, which may enhance their flocculation and adsorption performance. For example, chitosan/sodium alginate complex agents could achieve nearly 100% color/turbidity removal rates in sewage water under optimal conditions [13]. Carboxymethyl chitosan (CC), a water-soluble derivative of chitosan [14], and sodium alginate (SA), a polysaccharide extracted from brown seaweed [15], are two clarification agents widely recognized for their strong adsorption capacities. These properties are mainly attributed to the presence of functional groups such as amino, carboxyl, and hydroxyl moieties [16,17]. Under acidic conditions, CC becomes cationic, while SA retains an anionic character, enabling the formation of polyelectrolyte complexes through electrostatic interactions. Such CC-SA complexes have shown synergistic effects and improved flocculation performance compared to individual components and have been successfully utilized in various environmental applications, including heavy metal removal [18] and microalga harvesting [19]. Their potential application in food systems, however, remains underexplored—particularly in the context of clarifying turbid fruit juices like TCHJ.

Therefore, the present study aimed to investigate the clarification efficiency of a CC-SA composite for TCHJ. The clarification performance was systematically evaluated by monitoring juice transmittance under different conditions, including varying adsorbent dosages, adsorption times, and temperatures. A Box–Behnken response surface methodology (RSM) was employed to optimize the process parameters. This study aimed to fill the gap in clarifying thawed citrus juices by applying a novel CC–SA composite material and optimizing its application parameters. The study also evaluated material reusability, contributing to green processing strategies in the beverage industry.

## 2. Materials and Methods

### 2.1. Materials

Carboxymethyl chitosan (CC) and calcium carbonate (CaCO_3_) were procured from Shanghai Macklin Biochemical Technology Co., Ltd. (Shanghai, China). Sodium alginate (SA) and calcium chloride were procured from Shanghai Aladdin Bio-Chem Technology Co., Ltd. (Shanghai, China). Glutaraldehyde was procured from China National Pharmaceutical Group Co., Ltd. (Beijing, China). Citrus changshan-huyou juice was obtained from Zhejiang Aijia Fruit and Vegetable Development Co., Ltd. (Changshan, Zhejiang). The juice was delivered in frozen form and stored at −20 °C until use. For clarification trials, the juice was thawed at 4 °C for 24 h to minimize structural damage and component degradation. Unless otherwise stated, other materials used in this research were purchased from China National Pharmaceutical Group Co., Ltd.

### 2.2. Preparation of Carboxymethyl Chitosan/Sodium Alginate Composite Material

A total of 1.0 g of CC was dissolved in 100 mL of deionized water. The mixture was heated in a microwave oven until slightly boiling and stirred rapidly with a glass rod until a clear solution was obtained. The solution was then stirred using a magnetic stirrer at 100 °C and 800 rpm for 1 h. Subsequently, 1.0 g of SA powder was gradually added to the hot CC solution under continuous stirring with a glass rod until complete dissolution and clarity were achieved. The mixture was then further stirred on a magnetic stirrer under the same conditions (100 °C, 800 rpm) for an additional 1 h. Following this, 1.0 g of CaCO_3_ powder was added to the above mixture and stirred with a glass rod until a uniform white solution formed. The mixture was subjected to ultrasonic treatment to ensure uniform dispersion, resulting in a stable suspension. Glutaraldehyde was then added to the system to a final concentration of 0.5% (*v*/*v*), and the mixture was stirred in a water bath at 80 °C for 3 h to initiate gel network formation. Once the temperature had dropped to room temperature and a resilient gel had formed, the gel was immersed in 0.1 M hydrochloric acid for 24 h to dissolve the embedded calcium carbonate, thereby generating a porous structure. The resulting gel was subsequently washed with deionized water until the pH of the washed water remained above 6.5 for at least 1 h.

To enhance its mechanical stability, the gel was soaked in a 2% (*w*/*v*) calcium chloride solution for 1 h to achieve additional ionic crosslinking between Ca^2+^ and the alginate matrix. Finally, the gel was rapidly frozen in liquid nitrogen for 1 h and dried using a lyophilizer (SCIENTZ-10N/A, NINGBO SCIENTZ, Ningbo, China) at −55 °C under 5.0 Pa pressure for 48 h to obtain the porous CC-SA composite adsorbent.

### 2.3. Scanning Electron Microscopy (SEM)

The freeze-dried CC-SA composite was manually pulverized into fine powder using a ceramic mortar and pestle to ensure uniform particle sizes for subsequent clarification experiments. The microstructures of the CC-SA powders were observed using a scanning electron microscope (SU-70, Hitachi, Tokyo, Japan). The samples were gently spread onto conductive double-sided adhesive tape and then sputter-coated with a thin layer of gold prior to imaging.

The mean diameter of powdery microcapsules in the obtained pictures was calculated through ImageJ software (version 1.53e, Bethesda, Rockville, MD, USA). ImageJ is a public image processing software tool based on Java. By calculating the length of pixels at a given scale, this software could accurately submit the diameters of individual microcapsules in the SEM images.

### 2.4. Fourier Transform Infrared (FTIR) Spectroscopy

Fourier transform infrared (FTIR) spectroscopy (Spectrum 100, PerkinElmer, Shelton, CT, USA) was employed to identify the functional groups present in the CC–SA adsorbent. Approximately 0.002 g of each sample was thoroughly mixed with 0.2 g of anhydrous KBr and finely ground using an agate mortar. The resulting mixture was then pressed into a thin pellet using a cylindrical mold. Spectral data were collected by averaging 32 scans over the wavenumber range of 400–4000 cm^−1^.

### 2.5. Simultaneous Thermal Analysis (STA)

Approximately 15 mg of the composite sample was analyzed using a simultaneous thermal analyzer (STA6000, PerkinElmer, Shelton, CT, USA). The sample was heated from 20 °C to 800 °C at a rate of 10 °C/min under a nitrogen flow of 10 mL/min. Thermogravimetric (TG) and differential scanning calorimetry (DSC) signals were recorded simultaneously to monitor weight changes and thermal behavior.

### 2.6. Evaluation of Different Clarification Methods for TCHJ

Centrifugation Method I. A 30 mL aliquot of TCHJ was transferred into a 50 mL centrifuge tube and centrifuged at 5000 rpm for 10 min at room temperature. The transmittance of the TCHJ was measured at a wavelength of 660 nm using a UV-Vis spectrophotometer (UV-3100, MAPADA, Shanghai, China).

Centrifugation Method II. A 30 mL sample of TCHJ was centrifuged at 5000 rpm for 10 min at 4 °C. The supernatant was collected, and its transmittance was measured by UV-Vis spectrophotometry.

Clarification with CC. A 30 mL portion of TCHJ was mixed with 20 mg of CC in a 50 mL centrifuge tube. After stirring at room temperature for 60 min, the sample was centrifuged at 5000 rpm for 10 min. The transmittance of the supernatant was then measured.

Clarification with SA. A 30 mL TCHJ sample was treated with 20 mg of SA under the same conditions as above: 60 min stirring at room temperature followed by centrifugation at 5000 rpm for 10 min. The supernatant’s transmittance was determined.

Clarification with CC-SA Composite. A 30 mL aliquot of TCHJ was treated with 20 mg of the prepared CC-SA composite. After stirring at room temperature for 60 min, the sample was centrifuged at 5000 rpm for 10 min. Its transmittance was measured using a UV-Vis spectrophotometer.

All experiments in this research were performed in triplicate. The sample order for clarification trials was randomized to minimize potential systematic errors.

### 2.7. Clarification Performance Under Single-Factor Conditions

Effect of Dosage on Transmittance. Thirty-milliliter samples of TCHJ were placed into 50 mL centrifuge tubes, and different amounts of the CC-SA composite (10, 15, 20, 25, and 30 mg) were added. The mixtures were stirred at 30 °C for 60 min and then centrifuged at 5000 rpm for 10 min. The transmittance of each supernatant was recorded.

Effect of Adsorption Time on Transmittance. A fixed amount (20 mg) of the CC-SA composite was added to 30 mL of TCHJ. The samples were stirred at 30 °C for varying durations (40, 60, 80, 100, and 120 min), then centrifuged at 5000 rpm for 10 min. The supernatants were analyzed for their transmittance.

Effect of Adsorption Temperature on Transmittance. Thirty milliliters of TCHJ was treated with 20 mg of the composite material at different temperatures (20, 30, 40, 50, and 60 °C). After 80 min of stirring, each sample was centrifuged at 5000 rpm for 10 min, and its transmittance was measured.

### 2.8. Response Surface Optimization Design

A response surface methodology (RSM) was employed using Design-Expert software version 8.0.5 to optimize the clarification conditions. The transmittance of TCHJ was selected as the response variable, while three independent variables were investigated: the dosage of CC-SA composite (A, mg), adsorption time (B, min), and adsorption temperature (C, °C). Each variable was coded at three levels: −1 (low), 0 (medium), and +1 (high). The experimental design was based on a Box–Behnken model. The coded factors and their corresponding levels are listed in Table 1.

## 3. Results and Discussion

### 3.1. Scanning Electron Microscopy (SEM) Analysis

**Figure 1:**
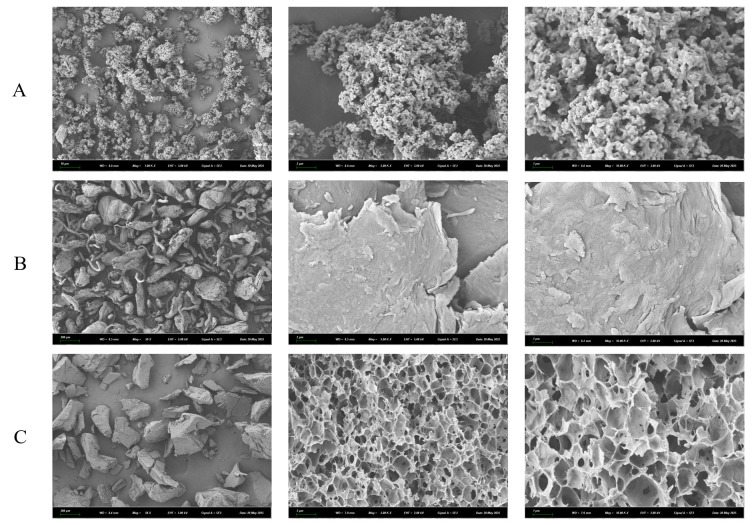
SEM images of carboxymethyl chitosan (**A**), sodium alginate (**B**), and carboxymethyl chi tosan/sodium alginate composite (**C**) materials.

The surface morphologies of CC, SA, and their CC-SA composite were observed using field emission SEM, as shown in Figure 1. The morphology of CC exhibited a granular structure with a loosely arranged and highly porous surface. At higher magnification, numerous small pores were observed (Figure 1A), which may facilitate the entrapment of suspended components in TCHJ through mechanisms such as bridging, net-trapping, or sweeping, thereby enhancing its clarification performance. SA showed a combination of fibrous and block-like structures with a distinctly wrinkled and irregular surface (Figure 1B), features that could effectively increase the contact area with target compounds and improve its adsorption efficiency. Figure 1C displays the surface morphology of the CC-SA composite. Compared with the individual components, the composite showed significantly altered structural features. It exhibited a predominantly block-like morphology with a denser network and larger pore sizes at higher magnification, suggesting improved potential for bridging and entrapment [20].

Based on the image analysis using ImageJ, the CC-SA composite showed an average pore diameter of 18.2 μm and a porosity of 97.6%. A high porosity is particularly beneficial for clarifying turbid huyou juice due to the associated enhanced surface area and improved mass transfer characteristics. The porous network provides abundant active sites for the physical entrapment and adsorption of suspended solids, polyphenols, and macromolecules such as pectins, proteins, and polysaccharides, which are primarily responsible for juice turbidity. Moreover, the interconnected pores facilitate efficient liquid infiltration and flocculation, allowing clarifying agents to interact more effectively with the haze-forming components.

Overall, the SEM images indicate that the CC-SA composite possesses a more favorable microstructure for capturing flocculent materials in TCHJ, which may enhance its clarification efficiency compared to the use of CC or SA alone.

### 3.2. Fourier Transform Infrared (FTIR) Spectroscopy Analysis

**Figure 2:**
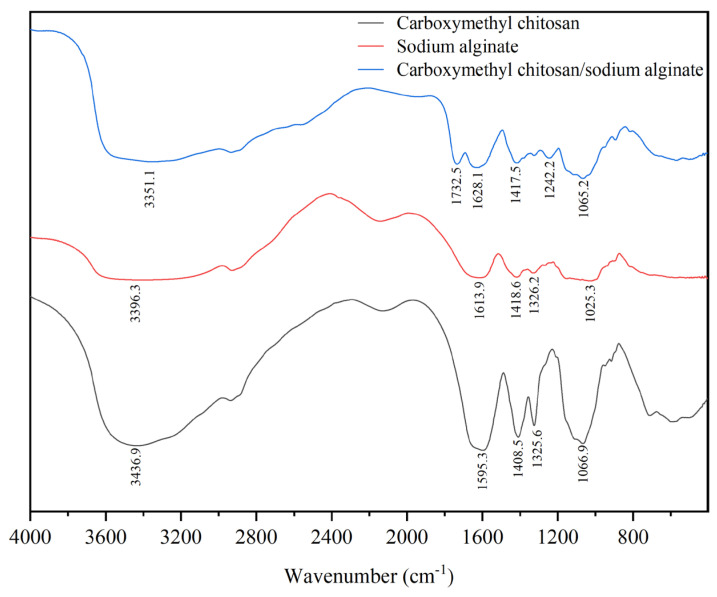
Infrared spectra of carboxymethyl chitosan (CC), sodium alginate (SA), and carboxymethyl chitosan/sodium alginate composite (CC-SA) materials.

The FTIR spectrum of CC (Figure 2) exhibited a broad absorption band centered at 3436.9 cm^−1^, attributed to overlapping O-H and N-H stretching vibrations. Characteristic peaks at 1595.3 cm^−1^ and 1408.5 cm^−1^ corresponded to the asymmetric and symmetric stretching vibrations of carboxylate groups (-COO-), respectively. The peak observed at 1325.6 cm^−1^ was assigned to C-N stretching, while the absorption at 1066.9 cm^−1^ was indicative of asymmetric C-O-C stretching vibrations associated with pyranose ring structures, reflecting the typical polysaccharide backbone of CC.

In the spectrum of SA, a broad peak at 3396.3 cm^−1^ was ascribed to O-H stretching. Peaks at 1613.9 cm^−1^ and 1418.6 cm^−1^ were assigned to the asymmetric and symmetric stretching vibrations of carboxylate groups, respectively. Additionally, the absorption band at 1025.3 cm^−1^ was associated with the C-O-C glycosidic linkage, a common structural feature of alginates [21].

The FTIR spectrum of the CC-SA composite showed notable spectral shifts, indicating strong intermolecular interactions. The O-H/N-H stretching band shifted to a lower wavenumber [22] (3351.1 cm^−1^), suggesting the formation of hydrogen bonds between CC and SA chains, which lowered the stretching vibration frequencies due to hydrogen bonding. A new peak emerged at 1732.5 cm^−1^, likely corresponding to the C=O stretching vibration of protonated carboxylic acid groups (-COOH), formed under acidic conditions [23]. This shift suggests the formation of ionic pairs through electrostatic interactions between carboxyl groups of SA and protonated amino groups of CC. Supporting this interpretation, the -COO- asymmetric stretching band exhibited a red shift of 14.2 cm^−1^, indicative of reduced electron density and enhanced electrostatic binding.

Moreover, the appearance of a new absorption peak at 1242.2 cm^−1^, assigned to C-O stretching, may reflect a conformational rearrangement of sugar rings triggered by molecular interactions between CC and SA chains [24]. These spectral changes collectively confirm the successful formation of a CC-SA composite with modified chemical environments and enhanced intermolecular interactions.

### 3.3. Simultaneous Thermal Analysis of Clarifying Materials

**Figure 3:**
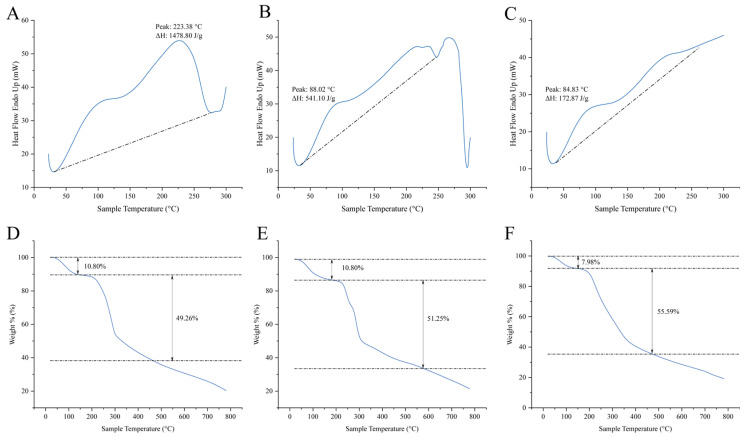
DSC thermal characteristic map of carboxymethyl chitosan (**A**), sodium alginate (**B**), and carboxymethyl chitosan/sodium alginate composite materials (**C**). TG curves of carboxymethyl chitosan (**D**), sodium alginate (**E**), and carboxymethyl chitosan/sodium alginate composite materials (**F**).

The DSC and TG curves of CC, SA, and the CC-SA composite material are shown in Figure 3. CC exhibited a major endothermic peak at 223.38 °C with an enthalpy change (ΔH) of 1478.8 J/g, corresponding to its crystalline melting behavior. In contrast, SA showed a lower enthalpy value of 541.1 J/g at 88.02 °C, indicating comparatively poor thermal stability. Notably, the melting point of the CC-SA composite decreased slightly to 84.83 °C (a reduction of 3.19 °C compared with SA), and its enthalpy value significantly dropped to 172.84 J/g—only 32% of that of SA. This reduction suggests strong intermolecular interactions between the two components, which may disrupt their original thermodynamic equilibrium and induce structural reorganization, thereby altering the thermal behavior of the composite.

The thermogravimetric analysis revealed two main weight-loss stages for all three materials. For CC (Figure 3D), the first stage occurred between 26.42 °C and 164.48 °C, corresponding to moisture loss, with a weight loss of 10.80%. The second degradation phase ranged from 164.48 °C to 440.06 °C, accounting for a 49.26% weight loss, leaving a residual mass of 20.32%. For SA (Figure 3E), initial degradation began at 29.97 °C with water loss (10.80%), followed by the second degradation stage starting at 194.5 °C and continuing to 554.4 °C, with a weight loss of 51.25% and a residual mass of 21.47%. In the case of the CC-SA composite (Figure 3F), the first weight-loss stage occurred between 27.79 °C and 85.81 °C due to moisture evaporation, with a weight loss of 7.98%. The second stage ranged from 85.81 °C to 459.94 °C, resulting in a 55.59% weight loss and leaving a residual mass of 13.26%.

Compared with the individual components, the CC-SA composite exhibited a significantly lower onset temperature for the second decomposition stage (85.81 °C), which was far below those of CC (164.48 °C) and SA (194.5 °C), while the decomposition endpoint fell between the two. Furthermore, the second-stage weight loss of the composite was higher, and the residual mass was considerably lower than those of CC and SA alone.

These results indicate that the CC-SA composite possesses distinct thermodynamic characteristics compared to its single-component counterparts. The enhanced thermal degradability may be attributed to the formation of a more thermally labile structure due to intermolecular interactions between CC and SA. Given the low decomposition temperature (~85 °C) of the CC–SA composite, its application is limited to processes below 70 °C or post-pasteurization addition. Direct application during high-temperature sterilization would risk structural degradation and loss of functionality.

### 3.4. Comparative Analysis of Different Clarification Methods for TCHJ

**Figure 4:**
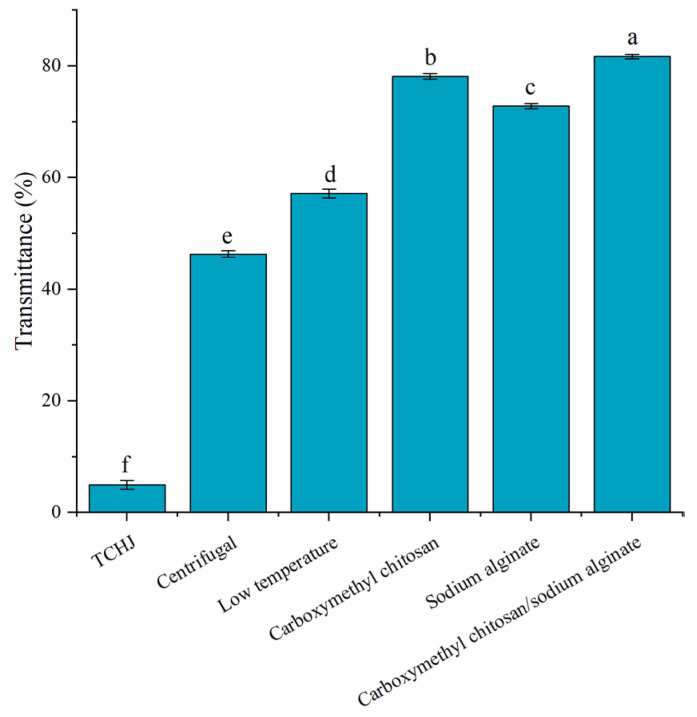
Effects of different methods on clarification of TCHJ. Different lowercase letters (a–f) indicate significant differences at *p* < 0.05.

As shown in Figure 4, the transmittance of untreated TCHJ was only 4.93%. However, all clarification treatments led to a significant increase in transmittance (*p* < 0.05). Among the tested methods, the use of the CC–SA composite exhibited the most effective clarification performance, elevating the transmittance to 81.67%, followed by CC alone (78.14%), SA alone (72.81%), low-temperature clarification (57.13%), and standard centrifugation (46.32%). Notably, the CC–SA composite outperformed both of its individual components, indicating a synergistic interaction between carboxymethyl chitosan and sodium alginate in enhancing juice clarity. These results suggest that the CC–SA composite provides better clarification performance (81.67%) for fruit juice than previously reported methods using bentonite (76.09%) [25] and conventional limed–carbonated (78.26%) [26] treatment.

This enhanced performance is likely attributable to the composite’s larger and more interconnected porous structure, as observed in previous characterizations. Such a structure may provide additional active sites for entrapping and adsorbing suspended flocculent particles within the TCHJ matrix, thereby facilitating more efficient removal of turbidity-causing substances and significantly improving overall juice clarity.

### 3.5. Single-Factor Analysis of CC-SA Composite Clarification Performance

To further investigate the clarification performance of the CC-SA composite in TCHJ, the effects of three key factors, the composite dosage, adsorption time, and adsorption temperature, were systematically evaluated. Each factor was studied individually to assess its influence on the transmittance of TCHJ and to optimize the clarification conditions.

**Figure 5:**
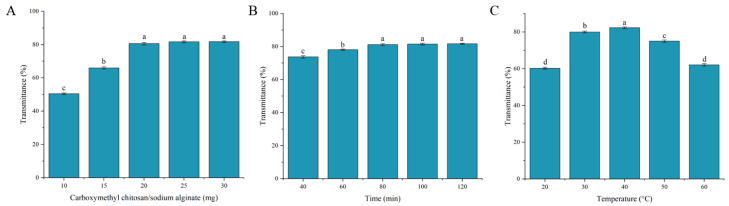
Effects of carboxymethyl chitosan/sodium alginate composite material dosage (**A**), adsorption time (**B**), and temperature (**C**) on clarification of TCHJ. Different lowercase letters (a–d) indicate significant differences at *p* < 0.05.

The transmittance of TCHJ increased progressively with a rising dosage of the CC-SA composite (Figure 5A). The highest transmittance (80.57%) was observed when the composite dosage reached 20 mg. Beyond this point, further increases in dosage resulted in negligible changes in transmittance. This plateau effect can be attributed to the finite concentration of turbidity-causing substances in TCHJ. Once these active particles have been sufficiently adsorbed, additional composite material no longer contributes significantly to clarification, indicating that the adsorption capacity for the suspended solids has reached saturation.

The transmittance of TCHJ increased significantly (*p* < 0.05) within the first 40 to 80 min of contact with the CC-SA composite (Figure 5B). After 80 min, the transmittance plateaued, reaching approximately 81%, and no further improvement in clarity was observed with extended adsorption time. This indicates that the adsorption capacity of the composite material was saturated at 80 min, beyond which additional contact time had minimal influence on the clarification performance.

The transmittance of TCHJ initially increased and then decreased with rising temperature (Figure 5C). A significant improvement (*p* < 0.05) was observed as the temperature increased from 20 °C to 40 °C, reaching the highest transmittance of 82.34% at 40 °C. This enhanced clarification efficiency at moderate temperatures may be attributed to the structural loosening of the CC-SA composite material at elevated temperatures, which increases the specific surface area and exposes more adsorption sites, thereby facilitating the removal of turbidity-causing compounds in TCHJ.

However, further increases in temperature led to a decline in transmittance. This may be due to the thermal instability of the composite material, resulting in reduced adsorption capacity. Such behavior aligns with the low enthalpy and limited thermal stability observed in the STA results discussed in Section 3.3. Additionally, excessive temperatures may cause the denaturation of turbidity-active proteins in TCHJ, leading to increased turbidity levels [27]. While protein denaturation is a plausible cause, this remains speculative. Alternatively, thermal collapse of the composite structure may reduce the available surface area. Future studies will quantify protein concentrations in the supernatant to verify denaturation effects.

### 3.6. Optimization of TCHJ Clarification Conditions Using Response Surface Methodology

To further optimize the TCHJ clarification process using the CC-SA composite material, a response surface methodology (RSM) was employed based on the results of the single-factor experiments. A three-factor, three-level Box–Behnken design was employed, with the composite dosage (A), adsorption time (B), and adsorption temperature (C) selected as the independent variables and transmittance (Y) as the response variable. A total of 16 experimental runs were carried out. The results of the RSM experiments are presented in Table 2, and the significance analysis of the regression model is shown in Table 3.

Using Design-Expert software, a second-order polynomial regression model was fitted to the experimental data (Table 2) to evaluate the effects of the composite dosage (A), adsorption time (B), and adsorption temperature (C) on TCHJ transmittance (Y). The resulting regression equation is as follows:

(1) *Y* = 80.03 + 11.13*A* − 0.6750*B* − 9.75*C* − 0.125*AB* + 1.93*AC* − 3.43*BC* − 7.41*A*^2^ − 1.76*B*^2^ − 11.61*C*^2^

According to the analysis of variance results (Table 3), the model was highly significant (*p* < 0.0001), while the lack-of-fit term was not significant (*p* = 0.1019 > 0.05), indicating that the model was well-fitted. Among the variables, the linear terms A (composite dosage) and C (adsorption temperature) showed extremely significant effects on transmittance (*p* < 0.0001). The interaction term BC had a significant effect (*p* < 0.001), and the quadratic terms A^2^ and C^2^ were also extremely significant (*p* < 0.0001). Other terms were not statistically significant (*p* > 0.05).

Based on the F-values, the factors influencing TCHJ transmittance in descending order were composite dosage > adsorption temperature > adsorption time.

Model evaluation parameters are presented in Table 4. The coefficient of determination R^2^ was 0.9905, and the adjusted R^2^ (R^2^Adj) was 0.9784, indicating a strong fit between the model and experimental data. The predicted R^2^ (R^2^Pred) was 0.8819, reflecting good predictive ability. The coefficient of variation (CV) was 2.72%, which is below the 5.0% threshold, indicating good model reproducibility. The signal-to-noise ratio (S/N = 30.34) was well above the minimum acceptable level of 4.0, confirming the model’s reliability.

**Figure 6:**
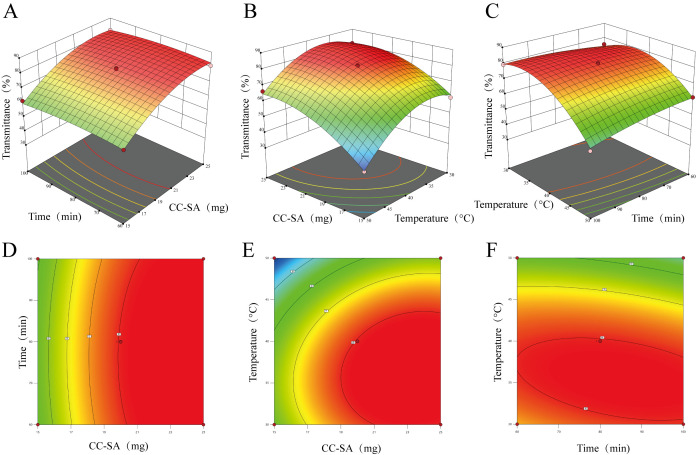
Response surface and contour plots illustrating the interaction effects between two variables on the clarification performance. (**A**,**D**) Interaction between CC-SA dosage and adsorption time; (**B**,**E**) Interaction between CC-SA dosage and adsorption temperature; (**C**,**F**) Interaction between adsorption time and adsorption temperature. (**A**–**C**) the red points represent that design points above predicted value; the pink points represent that design points below predicted value. (**D**–**F**) the closer to the central area (red), the higher the transmittance; the closer to the edge area (green), the lower the transmittance.

Response surface plots were constructed based on the regression equation using transmittance as the response variable. A steeper surface indicates higher sensitivity to changes in the corresponding variables, while more elliptical contour lines suggest a stronger interaction effect. As shown in Figure 6, the surface corresponding to the BC interaction (adsorption time and temperature) was relatively steep, and its contours were elliptical, indicating a significant interaction effect, consistent with the ANOVA results. Interactions between other factor pairs were not significant.

According to the optimization by Design-Expert, the optimal conditions for clarifying TCHJ using the CC-SA composite were a composite dosage of 21.85 mg, adsorption time of 80.30 min, and temperature of 37.78 °C, yielding a predicted transmittance of 84.84%. Considering operational feasibility, the optimal parameters were adjusted to a 22 mg dosage, 80 min adsorption time, and 38 °C temperature. Under these modified conditions, the actual measured transmittance of TCHJ reached 85.38%, which closely matched the predicted value, confirming the reliability and practicality of the developed model.

### 3.7. Reusability Analysis of the CC-SA Composite Material

**Figure 7:**
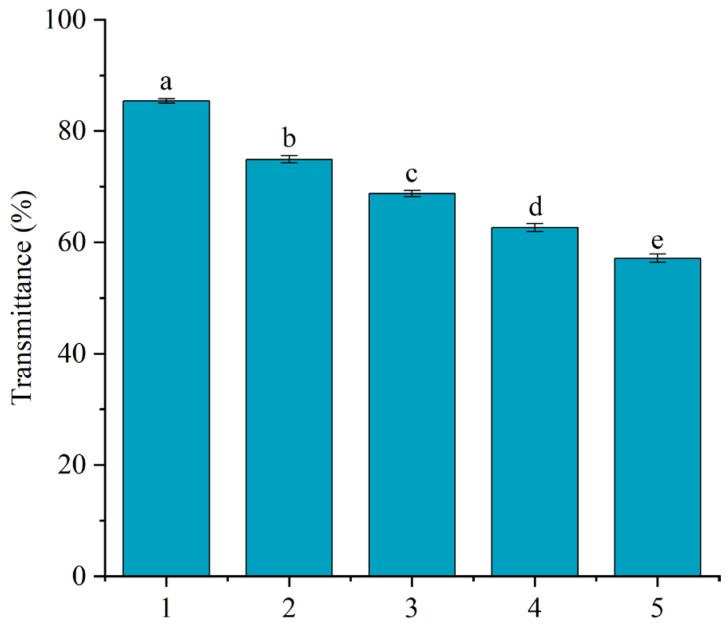
Reusability of carboxymethyl chitosan/sodium alginate composite materials for clarification of TCHJ. Different lowercase letters (a–e) indicate significant differences at *p* < 0.05.

As shown in Figure 7, a progressive decline in TCHJ transmittance was observed with repeated reuse of the CC-SA composite. This reduction reflects a gradual loss in adsorption efficiency, which became statistically significant (*p* < 0.05) after successive cycles. By the fifth reuse, the transmittance dropped to 57.13%, indicating a notable deterioration in clarification performance.

This reduction in performance may be attributed to the strong and broad-spectrum adsorption capacity of the CC-SA composite. In addition to binding proteins and small turbidity-causing molecules, the composite also adsorbs pectin and other colloidal substances. During the regeneration process, these components are not completely desorbed, leading to the accumulation of residues on the material. Consequently, the number of available adsorption sites diminishes with each cycle, resulting in a marked reduction in the composite’s clarification capacity after repeated use. A similar loss of adsorption capacity upon repeated use has been reported in biopolymer-based adsorbents [28,29]. The decline is likely due to the irreversible binding or accumulation of substances. In future studies, FTIR and elemental mapping will be used to assess residue accumulation.

## 4. Conclusions

In this research, a CC and SA composite material was characterized using scanning electron microscopy and Fourier transform infrared spectroscopy. The material was then applied to the clarification of turbid huyou juice, with transmittance used as the primary evaluation index to investigate the effects of individual factors—the composite dosage, adsorption time, and adsorption temperature—on clarification efficiency. A response surface methodology was employed to optimize the process, and the reusability of the composite was also assessed.

Electrostatic interactions between CC and SA likely promote the formation of a complex. Compared to CC or SA alone, the composite exhibited a larger and more porous network structure, enhancing its flocculation and entrapment capability. Among various clarification methods, using the CC-SA composite demonstrated the most effective performance in clarifying TCHJ, achieving a transmittance of 81.67%. Based on the response surface optimization and practical production considerations, the optimal clarification conditions were determined to be a composite dosage of 22 mg, adsorption time of 80 min, and temperature of 38 °C. Under these conditions, the transmittance reached 85.35%. Among all variables, composite dosage and adsorption temperature had the most significant effects on the clarification outcome.

## Figures and Tables

**Table 1:** Factors and levels used in Box–Behnken design.

Code	Variable	Unit	−1	0	1
*A*	Dosage	mg	15	20	25
*B*	Adsorption time	min	40	60	80
*C*	Adsorption temperature	°C	30	40	50

**Table 2:** Box–Behnken design and results.

Code	Composite Dosage (*A*)/mg	Adsorption Time(*B*)/min	Adsorption Temperature (*C*)/°C	Transmittance (*Y*)/%
1	25	40	60	81.1
2	15	40	100	61.4
3	25	40	100	81.4
4	15	30	80	60.1
5	25	30	80	80.5
6	15	50	80	38.2
7	25	50	80	66.3
8	20	30	60	75.6
9	20	30	100	79.2
10	20	50	60	61.5
11	20	50	100	51.4
12	20	40	80	81.3
13	20	40	80	78.8
14	20	40	80	81.4
15	20	40	80	80.9
16	20	40	80	79.1

**Table 3:** Analysis of variance for the fitted response surface model.

Source	Sum of Squares	df	Mean Square	*F* Value	*p* Value	Significance
*A*—dosage	990.13	1	990.13	269.84	<0.0001	***
*B*—time	3.64	1	3.64	0.9934	0.3521	
*C*—temperature	760.5	1	760.5	207.26	<0.0001	***
*AB*	0.0625	1	0.0625	0.017	0.8998	
*AC*	14.82	1	14.82	4.04	0.0844	
*BC*	46.92	1	46.92	12.79	0.009	**
*A* ^2^	231.35	1	231.35	63.05	<0.0001	***
*B* ^2^	13.08	1	13.08	3.56	0.101	
*C* ^2^	567.79	1	567.79	154.74	<0.0001	***
Model	2689.36	9	298.82	81.44	<0.0001	***
Residual	25.69	7	3.67			
Lack of Fit	19.43	3	6.48	4.14	0.1019	
Pure Error	6.26	4	1.57			
Total	2715.04	16				

**Note:** *p* < 0.01 (**), *p* < 0.001 (***).

**Table 4:** Variance correlation coefficients.

Parameter	Value	Parameter	Value
Standard Deviation	1.92	*R* ^2^	0.9905
Mean	70.52	*R* ^2^ *_Adj_*	0.9784
CV/%	2.72	*R* ^2^ *_Pred_*	0.8819
		S/N	30.3407

## Data Availability

The original contributions presented in this study are included in the article. Further inquiries can be directed to the corresponding author.

## Abbreviations

The following abbreviations are used in this manuscript:

TCHJ	Thawed cloudy huyou juice
CC	Carboxymethyl chitosan
SA	Sodium alginate
SEM	Scanning electron microscopy
FTIR	Fourier transform infrared spectroscopy
STA	Simultaneous thermal analysis
TG	Thermogravimetric
DSC	Differential scanning calorimetry
